# IRF4-activated TEX41 promotes the malignant behaviors of melanoma cells by targeting miR-103a-3p/C1QB axis

**DOI:** 10.1186/s12885-021-09039-1

**Published:** 2021-12-16

**Authors:** Yingna Zheng, Wu Zhou, Min Li, Ruixue Xu, Shuai Zhang, Ying Liu, Ying Cen

**Affiliations:** 1 Department of Dermatology, Henan Provincial People’s Hospital, People’s Hospital of Zhengzhou University, People’s Hospital of Henan University, 450003 Zhengzhou, Henan China; 2 Department of Dermatology, Xianyang Central Hospital, No.78, Renmin Road, 712000 Xianyang, Shaanxi China; 3 Department of Plastic and Burn Surgery, West China Hospital, West China School of Medicine, Sichuan University, 610041 Chengdu, Sichuan China

**Keywords:** Melanoma, TEX41, IRF4, miR-103a-3p, C1QB

## Abstract

**Background:**

Malignant melanoma is an aggressive skin cancer and a tumor of melanocytic origin. Recent studies have suggested that long non-coding RNAs (lncRNAs) play crucial regulatory roles in multiple malignancies, including melanoma. Testis expressed 41 (TEX41) is a relatively new lncRNA whose mechanism in melanoma remains vague.

**Aims:**

This study aimed to explore the role and specific mechanism of TEX41 in melanoma.

**Methods:**

The expression of genes involved in this study was determined by qRT-PCR. Functional assays were conducted to analyze the role of relevant genes in melanoma cells. The interaction between TEX41 promoter and IRF4 as well as the relationship among TEX41, miR-103a-3p and C1QB was verified by mechanism assays.

**Results:**

IRF4 up-regulated TEX41 at the transcriptional level in melanoma cells. TEX41 knockdown hindered melanoma cell proliferation, migration and invasion while promoting cell apoptosis. TEX41 bound to miR-103a-3p and regulated C1QB. The suppressive impact of TEX41 depletion on melanoma cell malignant behaviors could be counteracted by miR-103a-3p inhibition or C1QB overexpression. Moreover, IRF4 could facilitate melanoma cell growth via up-regulating C1QB.

**Conclusions:**

IRF4-activated TEX41 sequestered miR-103a-3p and modulated C1QB to promote melanoma cell malignant behaviors, for which TEX41 might be regarded as a potential therapeutic target for melanoma.

**Supplementary Information:**

The online version contains supplementary material available at 10.1186/s12885-021-09039-1.

## Background

Malignant melanoma, derived from melanocytes [1], is a type of aggressive cancer that occurs on the body surface or in internal organs [2]. Melanoma is characterized with distant metastasis through blood and lymph vessels, and poor prognosis [3]. Moreover, the incidence and mortality rates of melanoma have also increased in recent years [4], making the early diagnosis and timely treatment of melanoma particularly important. Therefore, it is necessary to study the molecular mechanism of melanoma development, so as to provide new targets for melanoma clinical treatment.

Multiple studies have shown that the abnormal expression of long non-coding RNAs (lncRNAs) is involved in the occurrence and development of tumors and diseases, including melanoma [5–7]. For example, lncRNA FOXD3-AS1 has been found to promote the proliferation, invasion and migration of cutaneous malignant melanoma by regulating miR-325/MAP3K2 [8]. Aside from that, LINC0638 has been revealed to be associated with local recurrence of melanoma [9]. Based on published reports, lncRNA TEX41 is a new research target involved in a variety of cancers by sponging different miRNAs [10]. For example, TEX41 promotes tumorigenesis via HPV integration [11]. The TEX41/miR-340/COMMD6 axis promotes the development of head and neck squamous cell carcinoma [12]. However, the role of TEX41 in melanoma has not been studied.

Our study aims to explore the function of TEX41 and uncover its potential mechanism in melanoma cells.

## Methods

### Cell culture

Melanoma cell lines (A375, WM35, A2058, SK-MEL-2) and normal epidermal melanin cell line (HEMa-LP) were used in this study. A375, A2058 and SK-MEL-2 cell lines were obtained from American Type Culture Collection (ATCC; Manassas, VA, USA), and WM35 as well as HEMa-LP cell lines were purchased from Xuanke Biotechnology Co., Ltd. (Shanghai, China). A375, WM35 and A2058 cell lines were cultured in Dulbecco’s Modified Eagle’s medium (DMEM, Invitrogen, Carlsbad, CA, USA). SK-MEL-2 cell line was maintained in Eagle’s Minimum Essential Medium (EMEM; ZQ-303, Shanghai Zhongqiaoxinzhou Biotech, Shanghai, China). HEMa-LP cell line was incubated in Medium 254 (M254500, Gibco, Grand Island, NY, USA). All media were added with 10% fetal bovine serum (FBS; 10,099,141 C, Gibco), and all cells were cultured with 5% CO_2_ at 37 °C.

### Plasmid construction and transfection

Mimic-miR-103a-3p, mimic-NC, miR-103a-3p inhibitor, inhibitor-NC, pcDNA3.1-IRF4, sh-IRF4#1/2, pcDNA3.1-FOXD3, pcDNA3.1-FOXM1,pcDNA3.1-TEX41, sh-TEX41#1/2, pcDNA3.1-C1QB, pcDNA3.1-C1QB-MUT, pcDNA3.1 and corresponding negative controls (sh-NCs) were purchased from Realgene (Shanghai, China). According to the protocol, lipofectamine 2000 (Invitrogen) was applied to transfect plasmids into melanoma cells after they reached 60-80% confluence in 12/96-well plates.

### RNA isolation and quantitative real-time PCR (qRT-PCR)

The total RNAs from melanoma cells were extracted by using TRIzol reagent (R0016, Beyotime, Shanghai, China). QuantiTect Reverse Transcription Kit (QIAGEN, Hilden, Germany) was used to obtain cDNA by reverse transcription. The SYBR PrimeScript RT-PCR kit (RR037A, Takara, Japan) was applied for RNA quantification. The endogenous controls of nucleus and cytoplasm were U6 and GAPDH, respectively. Also, the expression levels of RNAs were measured through utilizing 2^−∆∆Ct^ method. All primer sequences used in this study were displayed in Supplementary Table 1.

### Chromatin immunoprecipitation (ChIP)

A375 and SK-MEL-2 cells were fixed in 1% formaldehyde for 30 min, and the DNA was cut by sonication into fragments with an average fragment size of 500 ~ 1000 bp at room temperature. FOXD3 antibody, FOXM1 antibody and IRF4 antibody were used for chromatin immunoprecipitation, with IgG antibody as negative control. The purified chromatin precipitated by antibodies was quantified by qRT-PCR through using PowerUp™ SYBR® Green Master Mix (Life Technologies, Grand Island, NY, USA).

### Luciferase reporter assay

The wild-type sequences of TEX41 (TEX41-Wt), mutant sequence of TEX41 (TEX41-Mut), C1QB-Wt and C1QB-Mut were cloned into pmirGLO dual-luciferase vector respectively. TEX41-promoter-Wt and TEX41-promoter-Mut were inserted into pGL3 vector. The abovementioned pmirGLO plasmids were co-transfected into cells with mimic-NC or mimic-miR-103a-3p into melanoma cell lines. And pGL3 plasmids were co-transfected with pcDNA3.1 or pcDNA3.1-IRF4. Lipofectamine 2000 (Invitrogen) was applied for plasmid transfection. After 48 h, the luciferase activity was detected by Dual-Luciferase Reporter Assay System (Beyotime).

### Cell Counting Kit-8 (CCK-8) assay

According to manufacturer’s protocol, CCK-8 kit (Beyotime) was used to assess the proliferation of transfected melanoma cells. At first, cells were cultured in 96-well plates added with 10 µL CCK-8 solution for 2 h at 37 °C. The microplate reader was used to determine the optical density value at 450 nanometers.

### Colony formation assay

Transfected A375 and SK-MEL-2 cells were cultured in plastic culture dishes (500 cells per dish) with 5% CO_2_ at 37 °C for two weeks. Afterwards, the cells were washed with PBS (C0221A, Beyotime) twice, fixed with methanol for 10 min, and stained with crystal violet (C0121-100ml, Beyotime) for 30 min. Colonies were counted manually.

### 5-ethynyl-20-deoxyuridine (EdU) assay

EdU assay was conducted to measure cell growth. Transfected A375 and SK-MEL-2 cells were cultured in DMEM in 24-well plates. After the fixation in 4% paraformaldehyde, EdU (Sigma-Aldrich, Shanghai, China) was added to stain the cells. DAPI (Sigma-Aldrich) was used to label the cell nuclei. Finally, the laser scanning microscope was used to observe the treated cells.

### Transwell assay

Cell migration was detected by transwell assay in transwell chamber (3450, Corning, NY, USA). The upper layer of the chamber was added with cell suspension without FBS, while the lower chamber with complete medium. Each chamber was washed with PBS twice, followed by fixation by methanol at room temperature for 60 min. The crystal violet was used to stain the cells that had migrated to the lower chamber. The stained cells were observed and photographed under the microscope. Matrigel (356,234, BD Bioscience, NJ, USA) was used in the upper layer of the chamber for cell invasion assay, and the other steps were exactly the same as in the cell migration experiment.

### Transferase-mediated dUTP nick end labeling (TUNEL) assay

Cell Death Detection Kit (Sigma-Aldrich) was used to detect cell apoptosis in light of manufacturer’s instructions. DAPI was applied to stain the nucleus of transfected melanoma cells that were cultured in 6-well plates. Fluorescence microscopy (XSP-63B, Shanghai optical instrument factory, Shanghai, China) was adopted to capture the images of stained cells.

### Flow cytometry assay

Transfected A375 and SK-MEL-2 cells were cultured in 6-well plates. Annexin V-FITC/PI double staining kit (Invitrogen) was used to stain the cells for 15 min in dark environment. Next, cell apoptosis rate was detected with a flow cytometer (BD Biosciences, Franklin Lakes, NJ, USA).

### Fluorescent in situ hybridization (FISH) assay

The RNA FISH Kit (C10910, Ribobio, Guangzhou, China) was utilized to detect subcellular distribution in A375 and SK-MEL-2 cells. The experimental procedure was carried out as previously reported [13]. Cells were first incubated with FISH probes. DAPI was used to stain the nucleus. High resolution pictures were obtained by using laser scanning confocal microscope (Smart zoom5, Zeiss, Germany).

### Nucleo-cytoplasmic separation assay

Nucleo-cytoplasmic separation experiment was conducted by utilizing Cytoplasmic & Nuclear RNA Purification Kit (Norgen, Belmont, W.V, USA). The expression of TEX41 in cytoplasm and nucleus of A375 and SK-MEL-2 cells was tested by qRT-PCR with GAPDH/U6 as the cytoplasm/nucleus control.

### RNA binding protein immunoprecipitation (RIP) assay

In line with the manufacturer’s protocol, EZMagna RIP Kit (Shanghai Haoran Bio Technologies, Shanghai, China) was utilized for this assay. RIP lysis solution was added to lyse A375 and SK-MEL-2 cells. Next cell lysates were co-cultured with the magnetic beads (88,802, Thermo Fisher Scientific, Rockford, IL, USA) and antibody against Ago2 or IgG. IgG was used as a negative control. Finally, the purified RNA was analyzed by qRT-PCR.

### RNA pull down assay

Biotinylated (Bio)-TEX41-Wt, Bio-TEX41-Mut and Bio-NC were synthesized firstly. Afterwards, biotin-labeled probes were added into the cell lysates of A375 and SK-MEL-2 to carry out RNA pull down experiment. Subsequently, magnetic beads (HY-K0208, MedChemExpress, NJ, USA) were put into the cell lysates to obtain RNA complex conjoined with magnetic beads. After 2-hour incubation, the beads were washed with buffer solution (S7899, Sigma-Aldrich, St. Louis, MO, USA). The enrichment of miR-103a-3p in RNA-RNA complex was detected by qRT-PCR.

### Bioinformatics analysis

The expression level of TEX41 was predicted by GEPIA (http://gepia.cancer-pku.cn/index.html) [14] and TCGA (https://www.cancer.gov/about-nci/organization/ccg/research/structural-genomics/tcga) [15]; transcription factors (TFs) of TEX41 and their binding sites were predicted by Human TFDB (http://bioinfo.life.hust.edu.cn/HumanTFDB#!/) [16] and JASPAR (http://jaspar.genereg.net/analysis) [16]. MiRNAs of TEX41, mRNAs of miR-103a-3p as well as the binding sites were predicted by starBase (http://starbase.sysu.edu.cn/) [17].

### Statistical analysis

SPSS 18.0 software was used to perform statistical analysis; mean ± standard deviation (SD) was used to display the statistical values and all experiments were conducted for three times. The differences between two or more groups were compared by Student’s t-test or analysis of variance (ANOVA). The difference was considered to be statistically significant when P < 0.05.

## Results

### LncRNA TEX41 is highly expressed in melanoma tissues and cells and is associated with poor prognosis

**Fig. 1:**
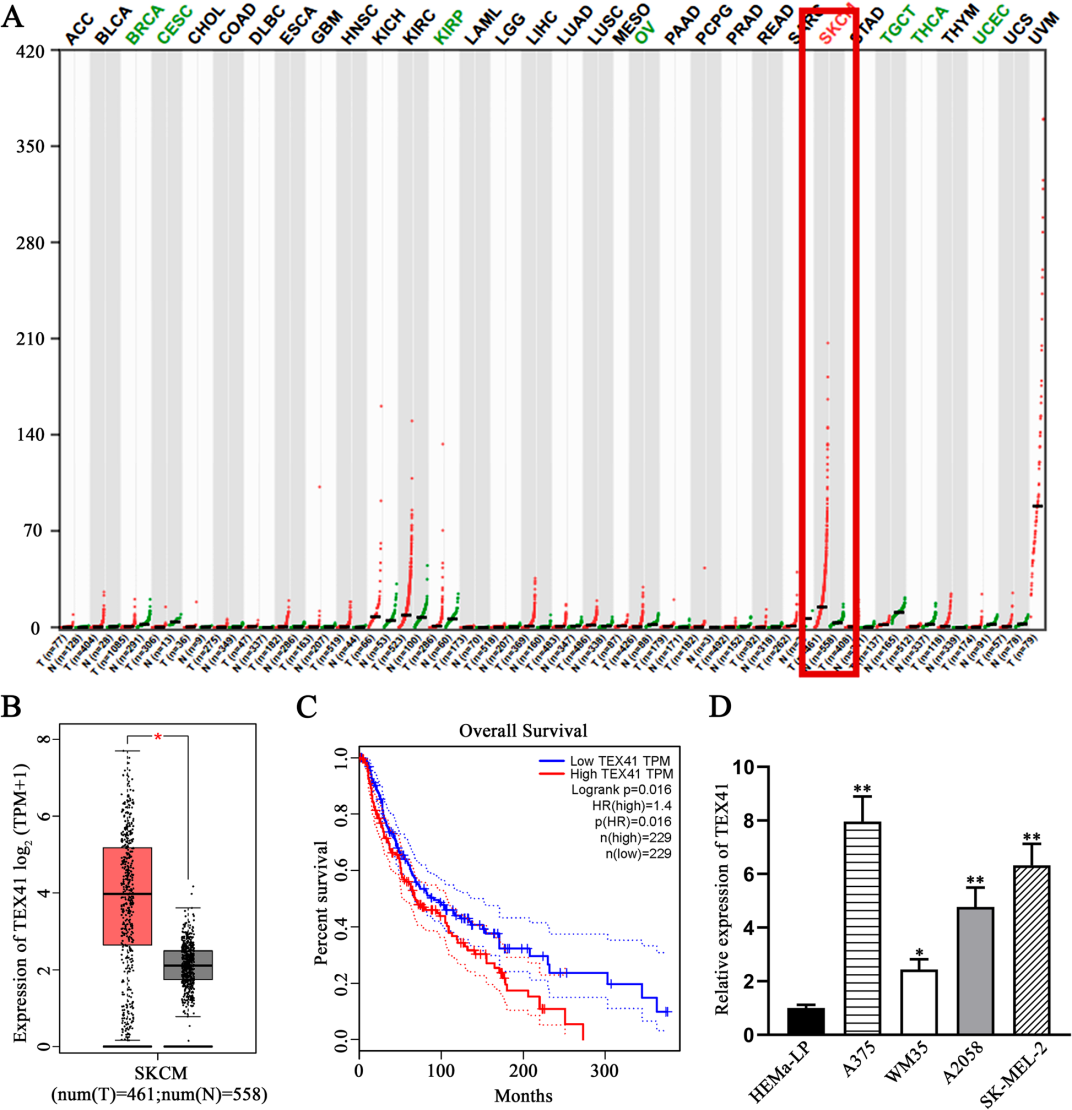
The expression of lncRNA TEX41 is high in melanoma cells and is associated with poor prognosis of melanoma patients. **A** The expression of TEX41 in tumor tissues and normal tissues was investigated by using GEPIA database. **B** The expression of TEX41 in SKCM tissues and normal skin tissues was obtained from GEPIA database. **C** The association between TEX41 expression and overall survival of SKCM patients was explored by GEPIA database. **D** The expression of TEX41 in melanoma cell lines and normal epidermal melanin cell line was examined through qRT-PCR.* P < 0.05, ** P < 0.01

To investigate the role of TEX41, we first used GEPIA database to figure out the expression of TEX41 in tumor tissues and normal tissues. The results showed that TEX41 was expressed at a higher level in skin cutaneous melanoma (SKCM) tissues than in normal skin tissues (Fig. 1 A-B). Moreover, survival analysis based on GEPIA showed that the overall survival of melanoma patients with high TEX41 expression was poorer than that of patients with low TEX41 expression (Fig. 1 C). These findings indicated that highly expressed TEX41 might be a factor associated with the survival of SKCM patients. Accordingly, we detected the expression of TEX41 in melanoma cell lines (A375, WM35, A2058, SK-MEL-2) and normal epidermal melanin cell line, HEMa-LP. The results of qRT-PCR analysis showed that TEX41 expression was higher in melanoma cell lines (especially in A375 and SK-MEL-2 cell lines) than in HEMa-LP cell line (Fig. 1D). In summary, TEX41 was over-expressed in SKCM tissues and melanoma cell lines. As the statistics illustrated that high TEX41 expression was associated with unfavorable prognosis of melanoma patients, the specific roles and mechanism of TEX41 in melanoma cells deserved further exploration.

### IRF4 activates the transcription of TEX41 in melanoma cells

**Fig. 2:**
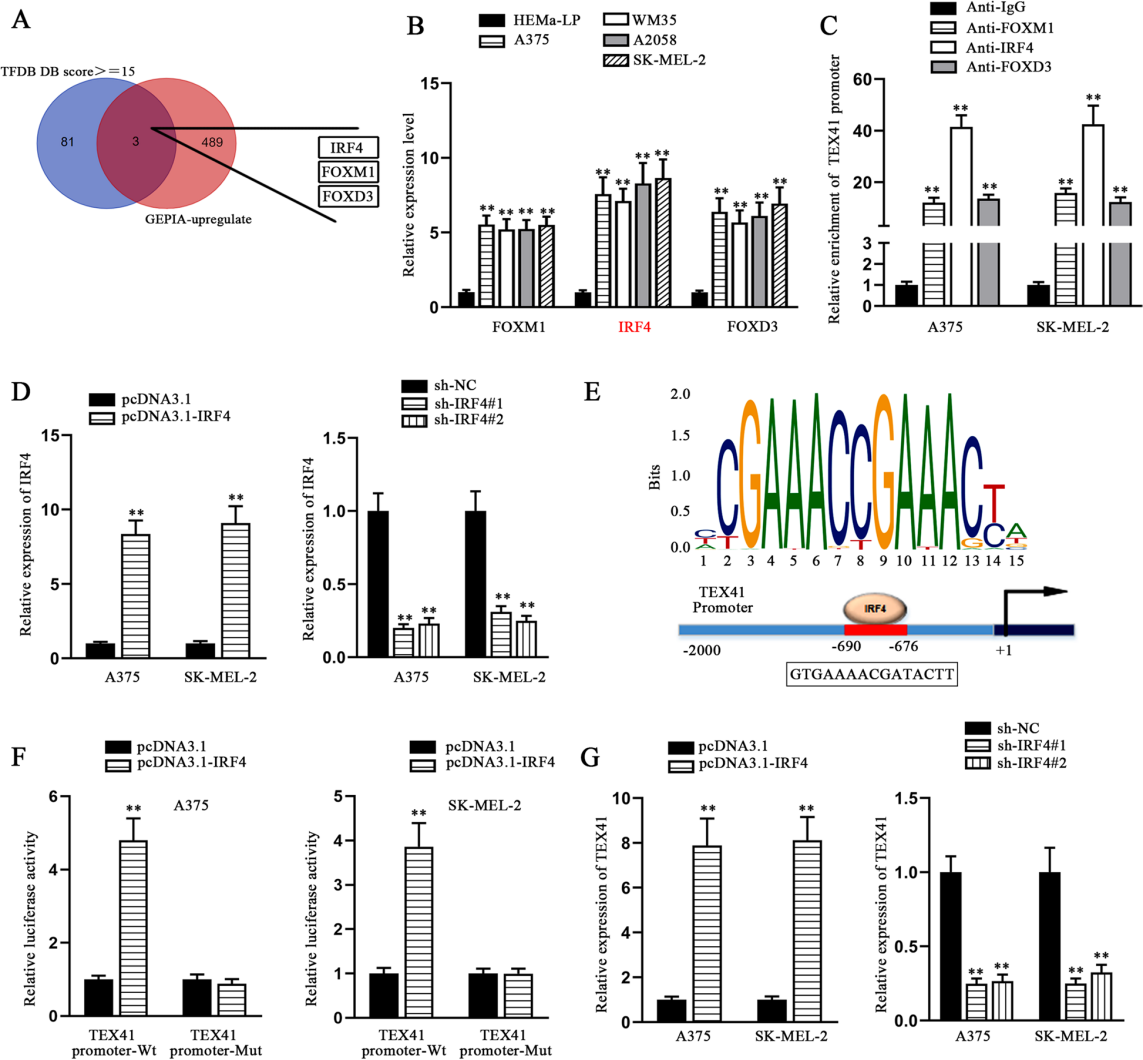
IRF4 activates the transcription of TEX41 in melanoma cells. **A** Three TFs (IRF4, FOXM1 and FOXD3) both potentially binding to TEX41 and up-regulated in SKCM were displayed as Venn diagram and selected out by bioinformatics analysis via Human TFDB (Predicted score >= 15) and GEPIA (log2 fold change > 2 and p < 0.05). **B** Expression levels of three candidate TFs in melanoma cells were detected via qRT-PCR. **C** ChIP assay was conducted to measure the affinity between the candidate TFs and TEX41 promoter. **D** The efficiency of IRF4 overexpression and knockdown was assessed by qRT-PCR. **E** Schematic diagram of binding sites between IRF4 and TEX41 promoter was predicted by Human TFDB and JASPAR. **F** Luciferase reporter assays were implemented to verify the binding affinity between IRF4 and TEX41 promoter. **G** The expression of TEX41 was detected by qRT-PCR in A375 and SK-MEL-2 cells with IRF4 augment or depletion. ** P < 0.01

TFs have been reported to work as crucial mediators of gene expression and their dysregulation may impact cancer prognosis [18]. Herein, we utilized bioinformatics tools to predict TFs that might bind to TEX41 and were up-regulated in SKCM tissues, and the searching outcomes were displayed as Venn diagram. Specifically, human TFDB was employed to screen out potential TFs that might combine with regulatory sequences of TEX41 under the indicated conditions: predicted score >= 15, upstream by 2000 bases and downstream by 100 bases. Then, we searched on GEPIA database to find the candidate genes at a high expression level in SKCM with the indicated conditions: log2 fold change > 2 and p < 0.05. Based on the overlap of two outcomes, three potential TFs of TEX41 stood out, including IRF4, FOXM1 and FOXD3 (Fig. 2 A). Next, the expression levels of three TFs were detected by qRT-PCR. The results showed that the three TFs all exhibited a higher expression in melanoma cell lines, compared with HEMa-LP. And IRF4 was more differentially expressed than the other two TFs (Fig. 2B). By means of ChIP assay, it was shown that IRF4 had a stronger affinity with the TEX41 promoter in A375 and SK-MEL-2 cells, compared with FOXM1 and FOXD3 (Fig. 2 C). The additional results of luciferase reporter assays demonstrated that there were no visible changes observed in TEX41-Wt group after the overexpression of FOXM1 and FOXD3, which excluded the possibility that FOXM1 or FOXD3 might take part in regulating the expression of TEX41 (Fig. S1A). After IRF4 was chosen, the overexpression efficiency of pcDNA3.1-IRF4 and knockdown efficiency of sh-IRF4#1/2 in A375 and SK-MEL-2 cells were detected by means of qRT-PCR. It turned out IRF4 could be remarkably overexpressed or reduced by pcDNA3.1-IRF4 or sh-IRF4#1/2 (Fig. 2D). Subsequently, JASPAR was applied to predict the possible binding sites between IRF4 and TEX41 promoter (Fig. 2E). The results of luciferase reporter assays displayed that IRF4 interacted with TEX41 promoter only when the binding sites were not mutated (Fig. 2 F). Moreover, qRT-PCR was conducted to detect the expression of TEX41 after melanoma cells were transfected with pcDNA3.1-IRF4 or sh-IRF4#1/2. The results demonstrated TEX41 could be elevated or diminished by IRF4 overexpression or knockdown, showing that IRF4 could positively regulate TEX41 expression (Fig. 2G). All the above results confirmed that IRF4 acted as a TF to activate the transcription of TEX41 in melanoma cells.

### Knockdown of TEX41 restrains malignant processes of melanoma cells

**Fig. 3:**
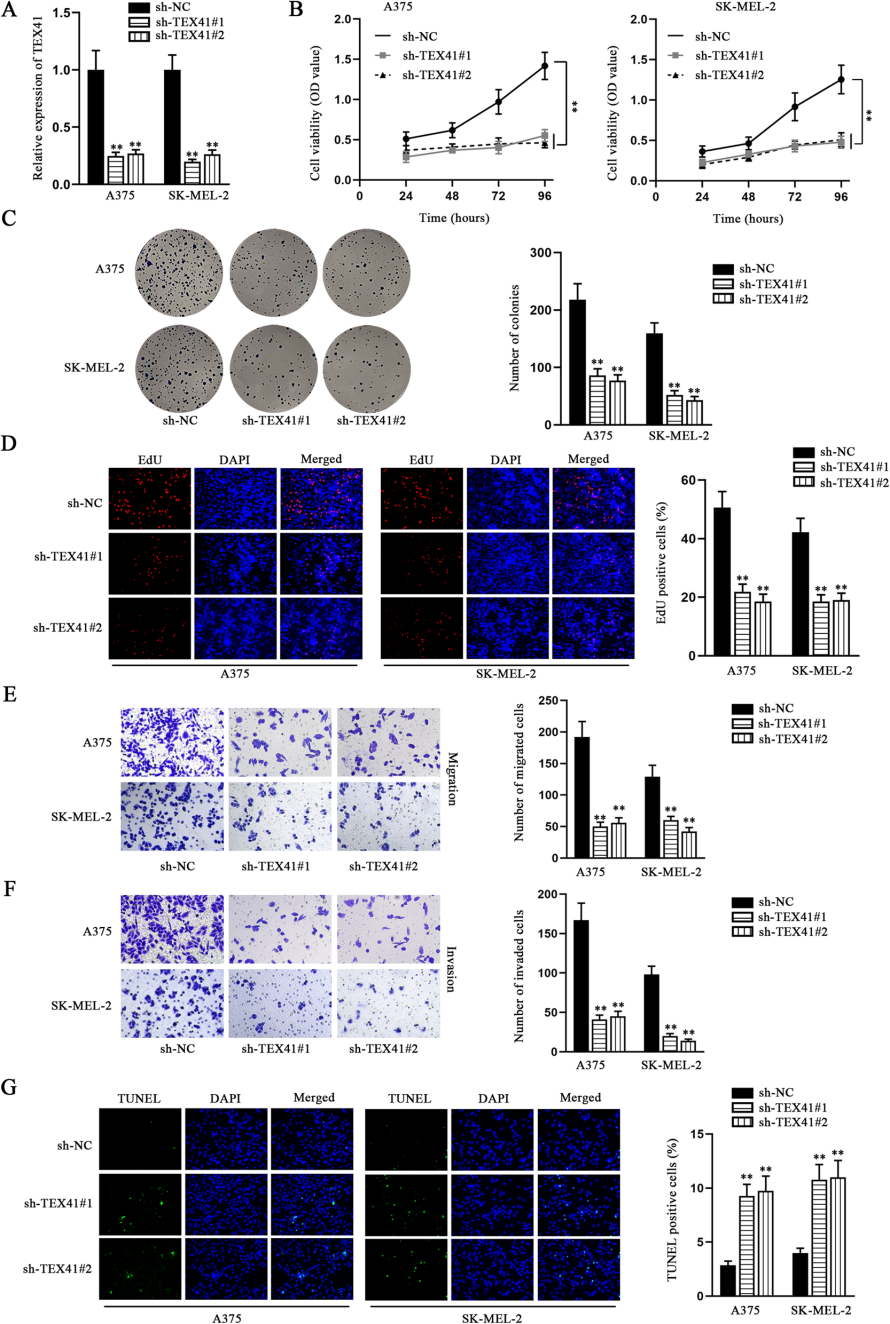
Knockdown of TEX41 affects the biological behaviors of melanoma cells. **A** QRT-PCR was conducted to detect TEX41 knockdown efficiency. **B** CCK-8 assays were utilized to detect the effect of TEX41 inhibition on melanoma cell viability. **C**-**D** Colony formation and EdU assays were carried out to measure melanoma cell proliferation in response to TEX41 knockdown. **E**-**F** Transwell assays were implemented to evaluate the migratory and invasive abilities of melanoma cells transfected with sh-TEX41#1/2. **G** TUNEL assay was used to measure apoptosis of melanoma cells. ** P < 0.01

To explore the effects of TEX41 on melanoma cells, we knocked down TEX41 in A375 and SK-MEL-2 cells (Fig. 3 A). In CCK-8 assays, the proliferation ability of melanoma cells with the transfection of sh-TEX41#1/2 was significantly impaired (Fig. 3B). The results of colony formation assay manifested that the colony formation ability of melanoma cells was weakened when the expression level of TEX41 was decreased (Fig. 3 C). Similarly, EdU assays also demonstrated that melanoma cell proliferation capacity was inhibited in response to TEX41 depletion (Fig. 3D). In addition, transwell assays were implemented to detect the migration and invasion ability of melanoma cells, and the results showed that the number of migrated and invaded cells was significantly reduced when the expression of TEX41 was lessened (Fig. 3E-F). Finally, TUNEL and flow cytometry assays were conducted to detect melanoma cell apoptosis, and it was found that the down-regulation of TEX41 accelerated the apoptosis of melanoma cells (Fig. 3G and S1B). Taken together, TEX41 served as an oncogene to facilitate the proliferation, migration, invasion, but hampered the apoptosis of melanoma cells.

### TEX41 directly binds to miR-103a-3p in melanoma cells

**Fig. 4:**
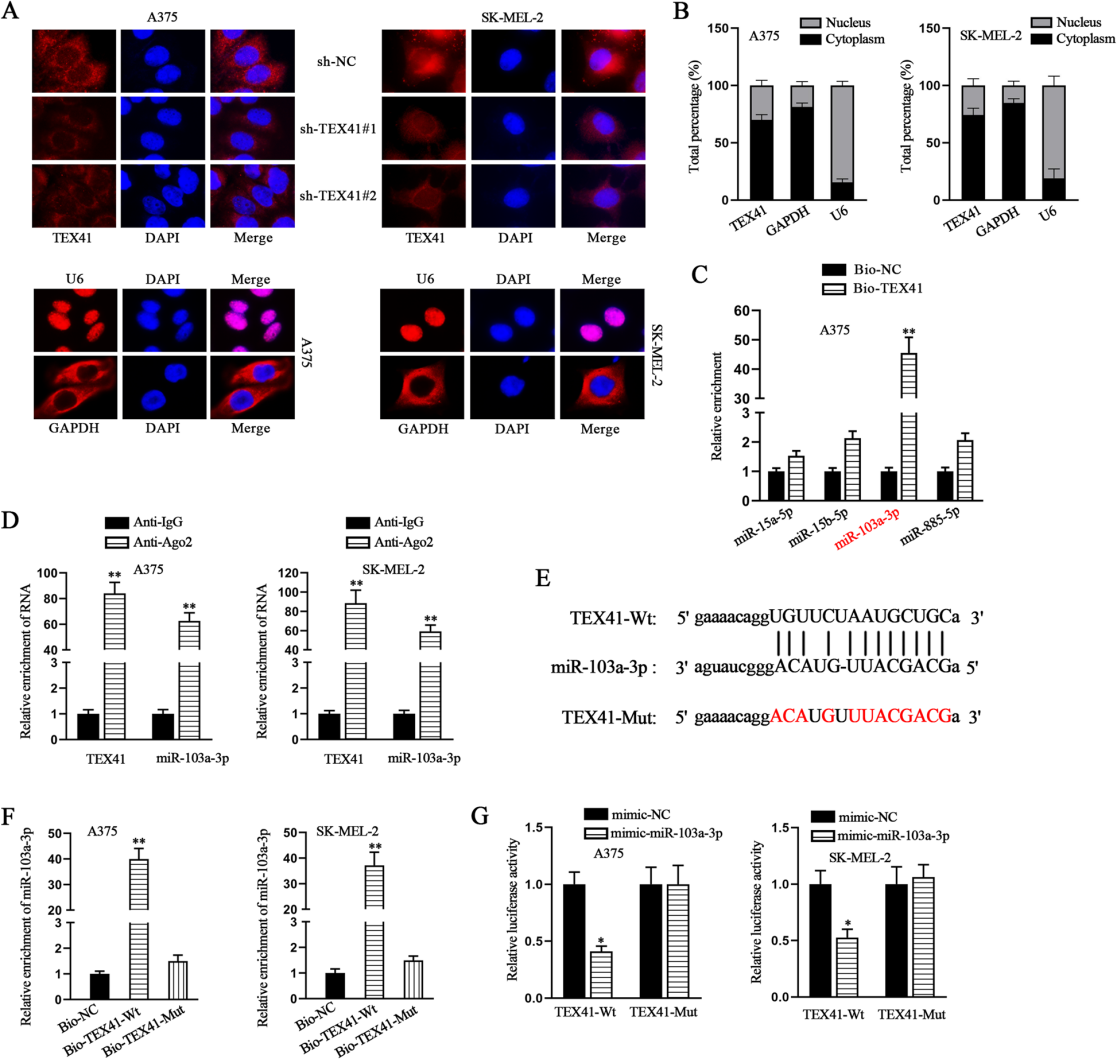
TEX41 directly sponges miR-103a-3p in melanoma cells. **A**-**B** The localization of TEX41 in melanoma cells was determined by FISH and Nucleo-cytoplasmic separation assays. **C** The enrichment of candidate miRNAs in Bio-TEX41 was assessed by RNA pull down assay. **D** The interaction between TEX41 and miR-103a-3p was confirmed by Ago2-RIP assay. **E** The binding sites between TEX41 and miR-103a-3p were predicted by starBase. **F**-**G** RNA pull down and luciferase reporter assays were performed to confirm the predicted binding sites between TEX41 and miR-103a-3p. * P < 0.05, ** P < 0.01

Knowing that the TEX41 had an oncogenic effect on melanoma cells, we further explored its regulatory mechanism. At the very beginning, the fluorescent images captured in FISH assay showed that TEX41 was mainly distributed in the cytoplasm. After TEX41 knockdown, cytoplasmic TEX41 was significantly reduced in melanoma cells (Fig. 4 A). Nucleo-cytoplasmic separation experiments, with U6 as the nuclear reference and GAPDH as the cytoplasmic reference, further verified the cytoplasmic distribution of TEX41 (Fig. 4B). Many reports have explained that cytoplasmic lncRNAs can act as competing endogenous RNAs (ceRNAs) by binding to miRNAs to modulate the targets of miRNAs at the post-transcriptional level [19, 20]. Thus we hypothesized that TEX41 might function through ceRNA network in melanoma cells. StarBase (http://starbase.sysu.edu.cn/) was used to predict potential miRNAs that might interact with TEX41 with the condition of Pan-Cancer >= 6, and four miRNAs (miR-15a-5p, miR-15b-5p, miR-103a-3p and miR-885-5p) were obtained. As shown in Fig. 4 C, the results of RNA pull down assay demonstrated that substantial miR-103a-3p could be pulled down by biotin-labeled TEX41 probe, for which the other three candidate miRNAs were excluded. Ago2 RIP assays further validated the interaction between miR-103a-3p and TEX41 (Fig. 4D). According to the above predictive outcomes of candidate miRNAs from starBase, we also obtained the potential binding sites between miR-103a-3p and TEX41 in Alignment column. We mutated binding sites in TEX41 for RNA pull down and luciferase reporter assays (Fig. 4E). RNA pull down results implied that miR-103a-3p was enriched in the biotin-labeled TEX41-Wt group rather than in the biotin-labeled TEX41-Mut group (Fig. 4 F). Then, the data from luciferase reporter assays proved that overexpressed miR-103a-3p significantly reduced the luciferase activity of TEX41-Wt in A375 and SK-MEL-2 cells, rather than that of TEX41-Mut (Fig. 4G). All these results above manifested that miR-103a-3p could bind to TEX41 in melanoma cells.

### C1QB is the target gene of miR-103a-3p in melanoma cells

**Fig. 5:**
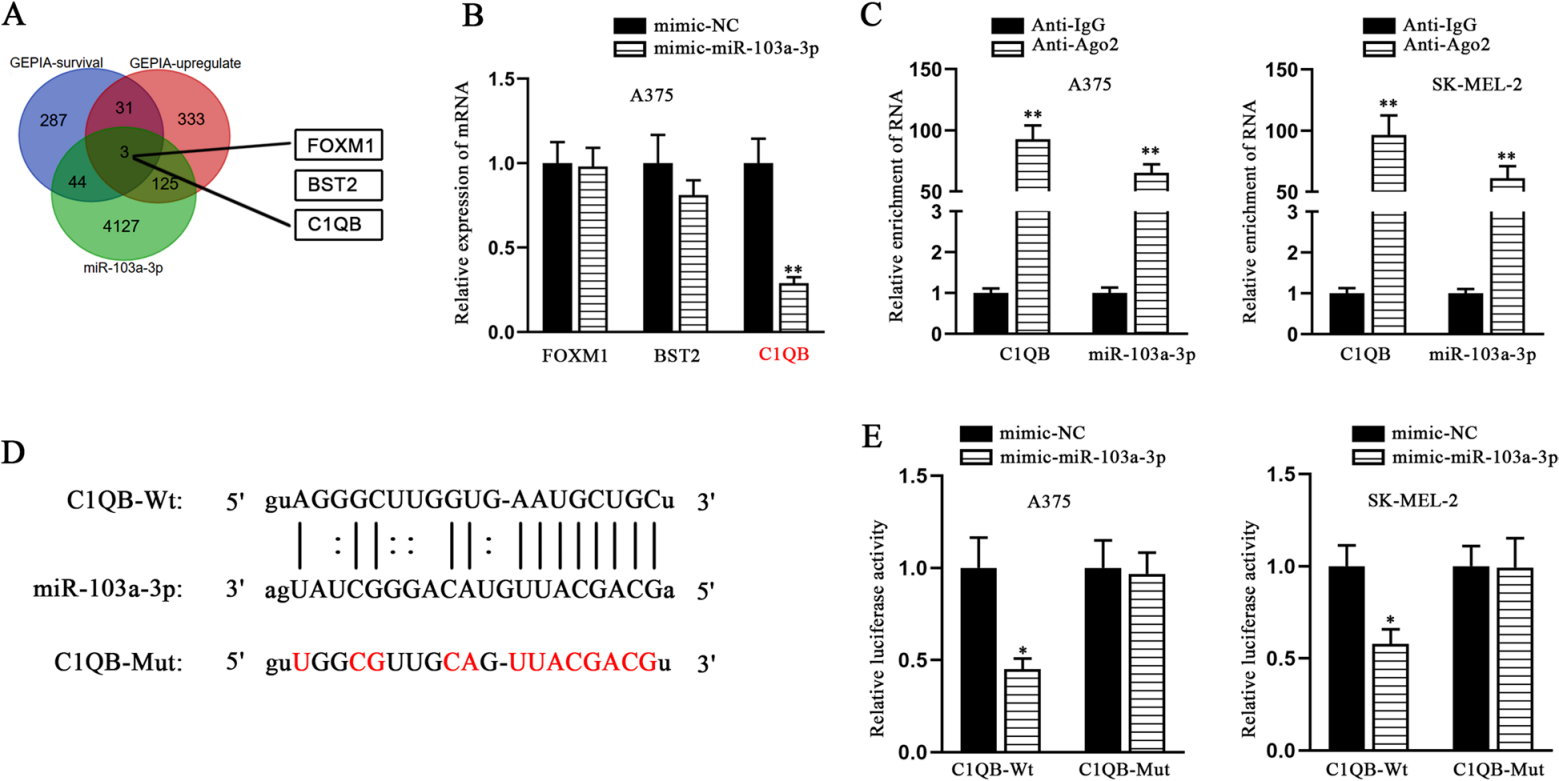
C1QB is the target gene of miR-103a-3p and plays a promoting role in melanoma cells. **A** Bioinformatics analysis via GEPIA-survival (p < 0.05), GEPIA-upregulate (log2 fold change < 2 and p > 0.05) and starBase singled out three candidate mRNAs (FOXM1, BST2 and C1QB) that may be the downstream targets of miR-103a-3p. **B** QRT-PCR was employed to assess the expression of the three mRNAs in A375 cells after the transfection with mimic-miR-103a-3p. **C** The combination between C1QB and miR-103a-3p was validated by Ago2-RIP assay. **D** StarBase was applied to project the potential binding sites of C1QB and miR-103a-3p. **E** Luciferase reporter assays were performed to validate the interaction between C1QB and miR-103a-3p on the potential binding sites. * P < 0.05, ** P < 0.01

Since miRNAs could exert their biological functions by mediating downstream mRNA, we tried to identify the potential downstream mRNAs of miR-103a-3p. GEPIA database was employed to output most differential survival genes (p < 0.05) and up-regulated genes (log2 fold change < 2 and p < 0.05) in SKCM. In addition, with no specific conditions, all candidate mRNAs targeted by miR-103a-3p were obtained from starBase. Considering the overlapped data from three predictions, we determined three candidate mRNAs (FOXM1, BST2 and C1QB) as the candidates (Fig. 5 A). To test if FOXM1, BST2 and C1QB were target genes of miR-103a-3p, qRT-PCR was conducted, and the results showed that when miR-103a-3p was up-regulated, C1QB was the most significantly down-regulated one in melanoma cells (Fig. 5B). In Ago2-RIP experiment, we verified the interaction between C1QB and miR-103a-3p as they were overtly enriched in anti-Ago2 (Fig. 5 C). StarBase website was employed to predict the potential binding sites between C1QB and miR-103a-3p, and the binding site in C1QB was mutated (Fig. 5D). The consequence of luciferase reporter assays showed that overexpressing miR-103a-3p reduced the luciferase activity of C1QB-Wt, but had little effects on that of C1QB-Mut (Fig. 5E). To sum up, C1QB was validated to work as the target gene of miR-103a-3p in melanoma cells.

### Knockdown of C1QB suppresses melanoma cell proliferation, migration and invasion, but promotes cell apoptosis

**Fig. 6:**
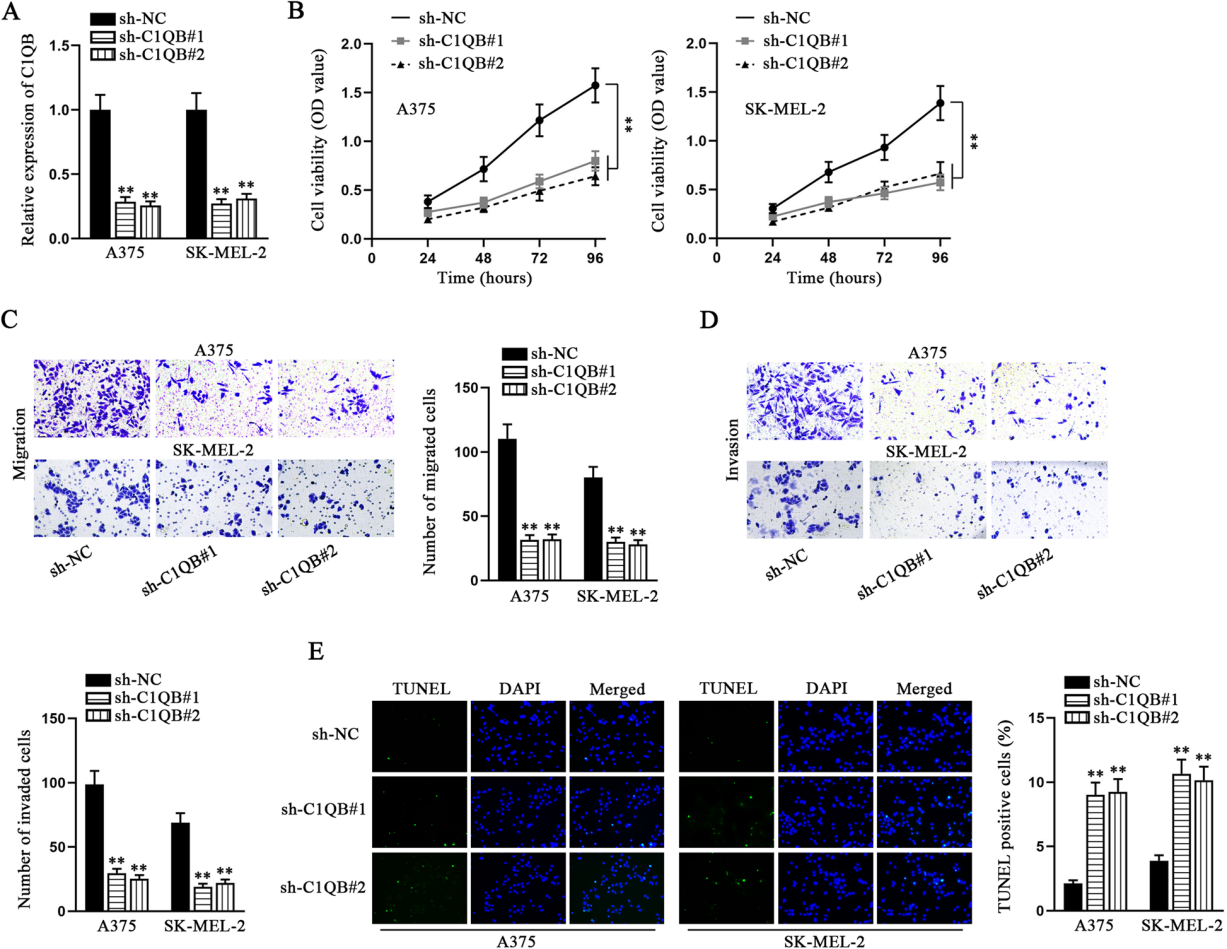
Knockdown of C1QB affects the biological behaviors of melanoma cells. **A** The knockdown efficiency of sh-C1QB#1/2 was assessed by qRT-PCR. **B** CCK-8 assays were carried to measure proliferation of melanoma cells upon C1QB reduction. **C**-**D** Transwell assays were implemented to evaluate the migration and invasion of melanoma cells in response to C1QB depletion. **E** TUNEL assay was used to measure apoptotic ability of melanoma cells with the condition of C1QB knockdown. ** P < 0.01

Prior to the exploration on the function of C1QB in melanoma cells, we first searched on GEPIA database to find the expression of C1QB in SKCM tissues and normal skin tissues. The data demonstrated the expression of C1QB was remarkably higher in SKCM tissues than that in normal skin tissues (Fig. S1C). Then, we performed qRT-PCR to detect the expression of C1QB in HEMa-LP and melanoma cells. The collected data indicated that C1QB was expressed at a higher level in melanoma cells, in contrast to HEMa-LP cells (Fig. S1D). A375 and SK-MEL-2 cells held the highest expression level of C1QB. Next, we successfully knocked down C1QB in A375 and SK-MEL-2 cells (Fig. 6 A). A series of functional experiments, including CCK8, transwell and TUNEL were carried out to respectively evaluate the influence of C1QB on proliferation, migration/invasion and apoptosis of melanoma cells. The outcomes indicated that knockdown of C1QB prominently dampened the proliferative, migratory and invasive abilities of melanoma cells (Fig. 6B-D), but accelerated melanoma cell apoptosis (Fig. 6E). Moreover, WM35 was selected for the following gain-of-function assays due to its relatively lower expression of C1QB compared with other melanoma cells. CCK-8, transwell migration and invasion assays were carried out using WM35 and HEMa-LP cells. The results suggested that the proliferative, migratory and invasive abilities of WM35 cells were stronger than those of HEMa-LP cells, and C1QB augment enhanced these abilities (Fig. S1E-G). In terms of TUNEL assay, the apoptosis of WM35 cells was weaker than that of HEMa-LP cells. Moreover, the apoptosis of WM35 and M3Ma-LP cells was restricted by C1QB overexpression (Fig. S1H). To conclude, these findings demonstrated that C1QB promoted proliferation, migration and invasion of melanoma cells, while inhibiting the cell apoptosis.

### C1QB overexpression and miR-103a-3p inhibition rescue the influence of TEX41 knockdown on melanoma cell behaviors

**Fig. 7:**
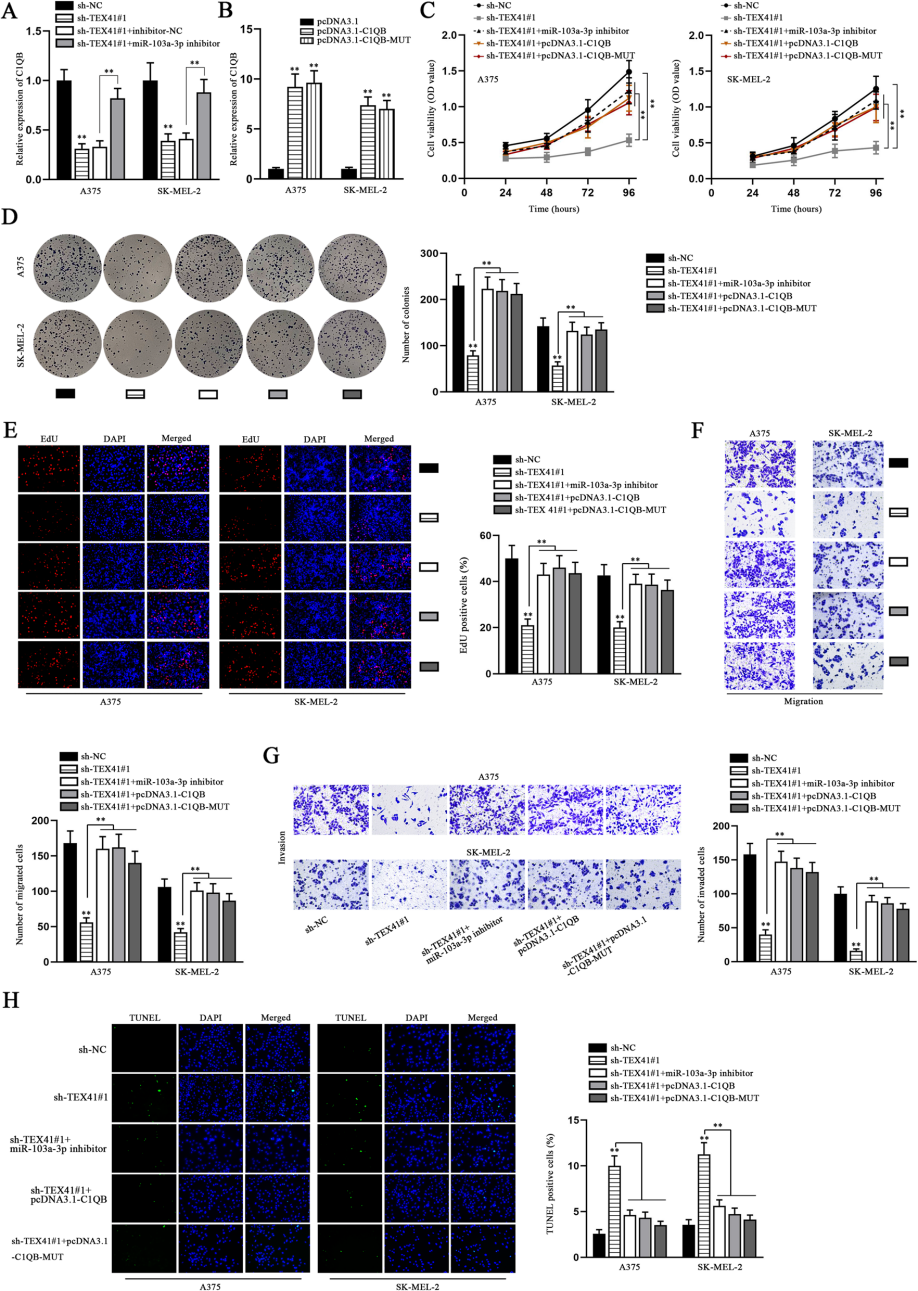
C1QB overexpression or miR-103a-3p inhibition rescues the influence of sh-TEX41 on melanoma cell behaviors. **A** The expression of C1QB was detected by qRT-PCR in melanoma cells transfected with different plasmids (sh-NC, sh-TEX41#1, SH-TEX41#1+inhibitor-NC or sh-TEX41#1+miR-103a-3p inhibitor). knockdown efficiency of miR-103a-3p inhibitor and **B** The overexpression efficiency of pcDNA3.1-C1QB/C1QB-Mut were determined by qRT-PCR. (**C**) CCK-8 assay was carried out to reveal the effect of C1QB/C1QB-Mut overexpression or miR-103a-3p down-regulation on inhibited melanoma cell viability induced by sh-TEX41#1. **D**-**E** Colony formation and EdU assays were carried out to examine whether pcDNA3.1-C1QB/C1QB-Mut or miR-103a-3p inhibitor could restore the inhibitory effect of sh-TEX41#1 on melanoma cell proliferation. **F**-**G** Transwell assays were done to verify the impact of C1QB/C1QB-Mut up-regulation or miR-103a-3p down-regulation on the restrained melanoma cell migration and invasion caused by TEX41 silencing. **H** Apoptosis of melanoma cells was detected via TUNEL assay under different conditions. ** P < 0.01

To further confirm whether TEX41 exerted its functions in melanoma cells via targeting miR-103a-3p/C1QB, we conducted a series of rescue experiments. Prior to that, qRT-PCR was conducted and it was found C1QB expression declined due to TEX41 knockdown but was restored by miR-103a-3p inhibition (Fig. 7 A). Next, the overexpression efficiency of pcDNA3.1-C1QB/C1QB-Mut was detected via qRT-PCR. The results showed that the expression level of C1QB was increased by transfection with pcDNA3.1-C1QB/C1QB-Mut plasmid (Fig. 7B). The results of CCK-8 assay showed that inhibition of miR-103a-3p or overexpression of C1QB could restore the suppressive effect of sh-TEX41#1 on cell viability (Fig. 7 C). Similarly, colony formation and EdU assays disclosed that when miR-103a-3p was diminished or C1QB was overexpressed, weakened proliferative ability of melanoma cells in response to sh-TEX41#1 transfection was significantly restored (Fig. 7D-E). Results of transwell assays manifested inhibition of miR-103a-3p or overexpression of C1QB rescued the reduced cell migration and invasion caused by TEX41 depletion (Fig. 7 F-G). TUNEL and flow cytometry assays were also performed to examine the apoptotic rate, and the augmented cell apoptosis induced by decreased TEX41 was recovered by miR-103a-3p inhibitor or pcDNA3.1-C1QB/C1QB-Mut (Fig. 7 H and S2A). In summary, the abovementioned findings suggested that TEX41 facilitated proliferation, migration and invasion of melanoma cells, but restrained the cell apoptosis by targeting miR-103a-3p/C1QB axis.

### C1QB overexpression reverses the inhibitory impact of IRF4 knockdown on melanoma cell growth

Aiming to verify the relationship between IRF4 and C1QB, we conducted qRT-PCR first. It turned out IRF4 depletion reduced the expression of C1QB, and IRF4 overexpression enhanced C1QB expression (Fig. S3A). Rescue assays were then performed to study the underlying mechanisms. As shown in Fig. S3B, cell proliferation was dramatically impeded after IRF4 knockdown, whereas co-transfection of pcDNA3.1-C1QB recovered the weakened cell proliferative ability. Data from transwell assays demonstrated suppressive effect of sh-IRF4#1 on cell migration and invasion was offset by C1QB overexpression (Fig. S3C-D). Moreover, the results of TUNEL assay reflected that enhanced cell apoptosis caused by IRF4 reduction was restored by up-regulation of C1QB (Fig. S3E). In brief, IRF4 raised C1QB expression in melanoma cells to facilitate cell growth.

## Discussion

As an aggressive form of cancer, melanoma has high recurrence and mortality rate [21]. With the research on the molecular mechanism of melanoma deepening, a large number of reports have confirmed that lncRNAs play a key role in the carcinogenesis of melanoma [22–24]. LncRNA TEX41, which has been investigated in cervical cancer and head and neck squamous cell carcinoma [11, 12], was chosen as the subject of our research. In this study, we aimed to investigate the regulatory role of TEX41 in melanoma. Firstly, TEX41 was found to display higher expression in melanoma tissues and cells than in normal skin tissues and normal epidermal melanin cells. And high TEX41 expression was correlated with poor prognosis of melanoma patients. Furthermore, we discovered that IRF4 stimulated the transcription of TEX41 and induced the aberrant up-regulation of TEX41 in melanoma cells. Functional experiments showed that knockdown of TEX41 inhibited melanoma cell proliferation, migration and invasion, while promoting melanoma cell apoptosis. Thus, the obtained data suggested that TEX41 played a promoting role in melanoma cells.

At present, a large number of studies have shown that lncRNAs could sponge miRNAs to regulate the function of mRNAs [23, 25]. In this study, a series of bioinformatics analyses and experiments indicated that miR-103a-3p was the downstream target of TEX41. MiR-103a-3p inhibition could recover the repressed malignant processes of melanoma cells induced by TEX41 knockdown. And it was found miR-103a-3p could bind to C1QB mRNA. Moreover, the expression level of C1QB was found to be negatively regulated by miR-103a-3p.

Referring to existing research work, C1QB has been reported to be negatively correlated with prognosis of gastric cancer [26]. According to other studies, C1QB is closely related to the brain tumor-induced epilepsy, whereas its specific role has not been discussed [27]. The expression of C1QB is correlated with the stage of renal cell carcinoma and poor prognosis of patients [28]. It has also been reported that C1QB is high-expressed in stage I and II melanoma patient samples. However, the function of C1QB in melanoma remains unexplored [29]. In this study, functional experiments revealed that down-regulation of C1QB inhibited melanoma cell proliferation, migration, invasion while promoting cell apoptosis. Rescue experiments further validated that overexpression of C1QB could counteract the effects of TEX41 depletion on the proliferation, migration, invasion and apoptosis of melanoma cells. Furthermore, IRF4 could modulate C1QB to affect melanoma cell growth. These results indicated the positive role of C1QB in affecting the biological functions of melanoma cells. To sum up, IRF4-elevated TEX41 promoted melanoma cell malignant behaviors via enhancing C1QB expression.

In summary, our study mainly illustrated the role of IRF4/TEX41/miR-103a-3p/C1QB axis in melanoma cells. Nonetheless, the underlying mechanism of C1QB in affecting biological behaviors of melanoma cells has not been discussed in this study, which will be the focus of our future study. The relation between TEX41 and melanoma has been studied for the first time. TEX41 may be a new potential biomarker that will be of great importance in the clinical diagnosis and treatment of melanoma.

## Conclusions

To sum up, TEX41 was highly expressed in melanoma cells, and its transcription activity was activated by IRF4. As a miR-103a-3p sponge and C1QB modulator, TEX41 promoted proliferation, migration and invasion of melanoma cells while repressing cell apoptosis. Therefore, we conclude that TEX41 might be a potential therapeutic target for melanoma.

## Supplementary Information

**Additional file 1: Figure S1.** (A) Luciferase reporter assays were carried out to verify the binding between TEX41 promoter and FOXM1/FOXD3. (B) Flow cytometry assays were used to measure apoptosis of melanoma cells upon TEX41 knockdown. (C) The expression of C1QB in SKCM tissues was obtained from GEPIA. (D) QRT-PCR was conducted to quantify the expression of C1QB in HEMa-LP and melanoma cell lines. (E-H) CCK-8, transwell as well as TUNEL assays were performed to evaluate the effect of C1QB augment on viability, migration, invasion and apoptosis of HEMa-LP and WM35 cells. * P < 0.05, ** P < 0.01.

**Additional file 2: Figure S2.** (A) Apoptosis of melanoma cells was measured by flow cytometry assays. ** P < 0.01.

**Additional file 3: Figure S3.** (A) The expression of C1QB in melanoma cells was quantified via qRT-PCR after IRF4 depletion/overexpression. In rescue assays, three groups were set: sh-NC, sh-IRF4#1 and sh-IRF4#1+pcDNA3.1-C1QB. (B) Colony formation assay was carried out to evaluate proliferation of melanoma cells under different conditions. (C-D) Cell migratory and invasive abilities were analyzed in transwell assays. (E) Cell apoptosis was detected via TUNEL assay. ** P < 0.01.

**Additional file 4: Supplementary Table 1.** Primer sequences involved in qRT-PCR were listed.

## Data Availability

Not applicable.

## Abbreviations

lncRNAs: Long non-coding RNAs
TEX41: Testis expressed 41
IRF4: Interferon regulatory factor 4
C1QB: Complement C1q B chain
ATCC: American Type Culture Collection
DMEM: Dulbecco’s Modified Eagle’s medium
EMEM: Eagle’s Minimum Essential Medium
FBS: Fetal bovine serum
qRT-PCR: Quantitative real-time PCR
CCK8: Cell Counting Kit-8
EdU: 5-ethynyl-20-deoxyuridine
TUNEL: Transferase-mediated dUTP nick end labeling
FISH: Fluorescent in situ hybridization
RIP: RNA immunoprecipitation
ChIP: Chromatin immunoprecipitation
IgG: Immunoglobulin G
ceRNA: Competing endogenous RNA
Wt: Wild-type
Mut: Mutant type

## References

[1] Long J, Pi X (2018). lncRNA-MEG3 Suppresses the Proliferation and Invasion of Melanoma by Regulating CYLD Expression Mediated by Sponging miR-499-5p. BioMed research international.

[2] Pavri SN, Clune J, Ariyan S, Narayan D (2016). Malignant Melanoma: Beyond the Basics. Plastic and reconstructive surgery.

[3] Abbas O, Miller DD, Bhawan J (2014). Cutaneous malignant melanoma: update on diagnostic and prognostic biomarkers. The American Journal of dermatopathology.

[4] Rastrelli M, Tropea S, Rossi CR, Alaibac M (2014). Melanoma: epidemiology, risk factors, pathogenesis, diagnosis and classification. In vivo (Athens. Greece).

[5] Yang G, Lu X, Yuan L (2014). LncRNA: a link between RNA and cancer. Biochimica et biophysica acta.

[6] Chi Y, Wang D, Wang J, Yu W, Yang J. Long Non-Coding RNA in the Pathogenesis of Cancers. Cells. 2019;8(9). 10.3390/cells8091015. 10.3390/cells8091015 PMC6770362 31480503

[7] Peng WX, Koirala P, Mo YY (2017). LncRNA-mediated regulation of cell signaling in cancer. Oncogene.

[8] Chen T, Yang Z, Liu C, Wang L, Yang J, Chen L (2019). Circ_0078767 suppresses non-small-cell lung cancer by protecting RASSF1A expression via sponging miR-330-3p. Cell proliferation.

[9] Xiao W, Yin A (2019). LINC0638 lncRNA is involved in the local recurrence of melanoma following surgical resection. Oncology letters.

[10] Coe EA, Tan JY, Shapiro M, Louphrasitthiphol P, Bassett AR, Marques AC (2019). The MITF-SOX10 regulated long non-coding RNA DIRC3 is a melanoma tumour suppressor. PLoS genetics.

[11] Li W, Qi Y, Cui X, Huo Q, Zhu L, Zhang A (2018). Characteristic of HPV Integration in the Genome and Transcriptome of Cervical Cancer Tissues. BioMed research international.

[12] Yang M, Huang W, Sun Y, Liang H, Chen M, Wu X (2019). Prognosis and modulation mechanisms of COMMD6 in human tumours based on expression profiling and comprehensive bioinformatics analysis. British journal of cancer.

[13] Ozemri Sag S, Yakut T, Gorukmez O, Gorukmez O, Ture M, Karkucak M (2015). Qualitative and quantitative evaluation of the BCR-ABL fusion gene in chronic myelogenous leukemia by flourescence in situ hybridization and molecular genetic methods. Genetic testing and molecular biomarkers.

[14] Tang Z, Li C, Kang B, Gao G, Li C, Zhang Z (2017). GEPIA: a web server for cancer and normal gene expression profiling and interactive analyses. Nucleic acids research.

[15] Wang Z, Jensen MA, Zenklusen JC (2016). A Practical Guide to The Cancer Genome Atlas (TCGA). Methods Molecular Biol.

[16] Chen HF, Wang JK (2010). [The databases of transcription factors.]. Yi chuan = Hereditas.

[17] Yang JH, Li JH, Shao P, Zhou H, Chen YQ, Qu LH (2011). starBase: a database for exploring microRNA-mRNA interaction maps from Argonaute CLIP-Seq and Degradome-Seq data. Nucleic acids research.

[18] Li R, Jiang S, Li W, Hong H, Zhao C, Huang X (2019). Exploration of prognosis-related microRNA and transcription factor co-regulatory networks across cancer types. RNA biology.

[19] Thomson DW, Dinger ME (2016). Endogenous microRNA sponges: evidence and controversy. Nature reviews Genetics.

[20] Zhou RS, Zhang EX, Sun QF, Ye ZJ, Liu JW, Zhou DH (2019). Integrated analysis of lncRNA-miRNA-mRNA ceRNA network in squamous cell carcinoma of tongue. BMC cancer.

[21] Lerner BA, Stewart LA, Horowitz DP, Carvajal RD (2017). Mucosal Melanoma: New Insights and Therapeutic Options for a Unique and Aggressive Disease. Oncology (Williston Park, NY).

[22] Yu X, Zheng H, Tse G, Chan MT, Wu WK (2018). Long non-coding RNAs in melanoma. Cell proliferation.

[23] Sun Z, Wu F, Yang Y, Liu F, Mo F, Chen J (2019). MiR-144-3p Inhibits BMSC Proliferation and Osteogenic Differentiation Via Targeting FZD4 in Steroid-Associated Osteonecrosis. Current pharmaceutical design.

[24] Sarkar D, Leung EY, Baguley BC, Finlay GJ, Askarian-Amiri ME (2015). Epigenetic regulation in human melanoma: past and future. Epigenetics.

[25] Qi X, Zhang DH, Wu N, Xiao JH, Wang X, Ma W (2015). ceRNA in cancer: possible functions and clinical implications. Journal of medical genetics.

[26] Jiang J, Ding Y, Wu M, Lyu X, Wang H, Chen Y (2020). Identification of TYROBP and C1QB as Two Novel Key Genes With Prognostic Value in Gastric Cancer by Network Analysis. Frontiers in oncology.

[27] Niesen CE, Xu J, Fan X, Li X, Wheeler CJ, Mamelak AN (2013). Transcriptomic profiling of human peritumoral neocortex tissues revealed genes possibly involved in tumor-induced epilepsy. PloS one.

[28] Yamada Y, Arai T, Kojima S, Sugawara S, Kato M, Okato A (2018). Regulation of antitumor miR-144-5p targets oncogenes: Direct regulation of syndecan-3 and its clinical significance. Cancer science.

[29] Luo Y, Robinson S, Fujita J, Siconolfi L, Magidson J, Edwards CK (2011). Transcriptome profiling of whole blood cells identifies PLEK2 and C1QB in human melanoma. PloS one.

