# Supplementary figures and images for: IRF4-activated TEX41 promotes the malignant behaviors of melanoma cells by targeting miR-103a-3p/C1QB axis
Source: BMC Cancer. 2021 Dec 16;21:1339. doi: 10.1186/s12885-021-09039-1 (PMC8680380; doi:10.1186/s12885-021-09039-1)

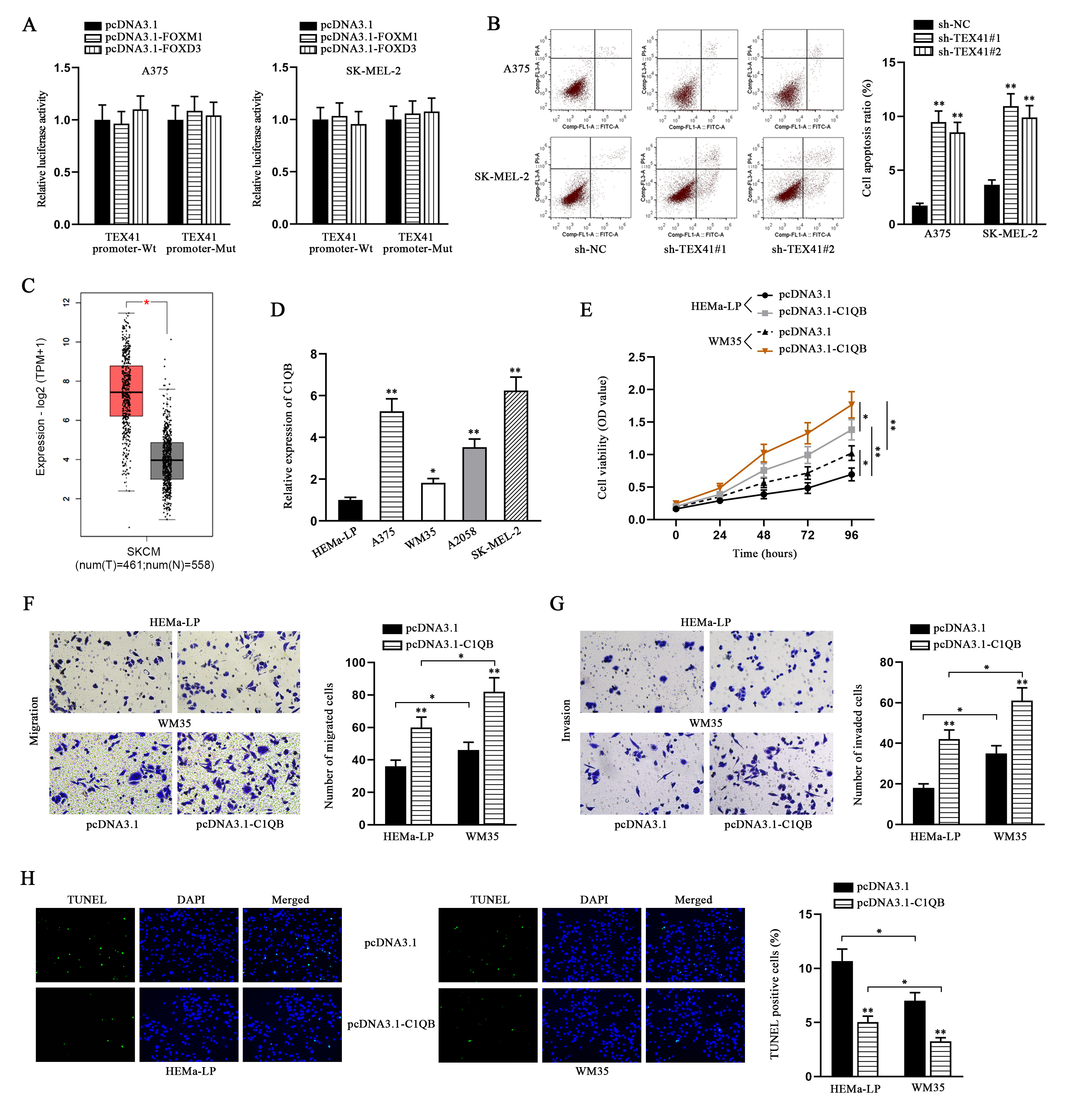

Supplement: Supplementary file 1 — Additional file 1: Figure S1. (A) Luciferase reporter assays were carried out to verify the binding between TEX41 promoter and FOXM1/FOXD3. (B) Flow cytometry assays were used to measure apoptosis of melanoma cells upon TEX41 knockdown. (C) The expression of C1QB in SKCM tissues was obtained from GEPIA. (D) QRT-PCR was conducted to quantify the expression of C1QB in HEMa-LP and melanoma cell lines. (E-H) CCK-8, transwell as well as TUNEL assays were performed to evaluate the effect of C1QB augment on viability, migration, invasion and apoptosis of HEMa-LP and WM35 cells. * P < 0.05, ** P < 0.01. [file 12885_2021_9039_MOESM1_ESM.tif]

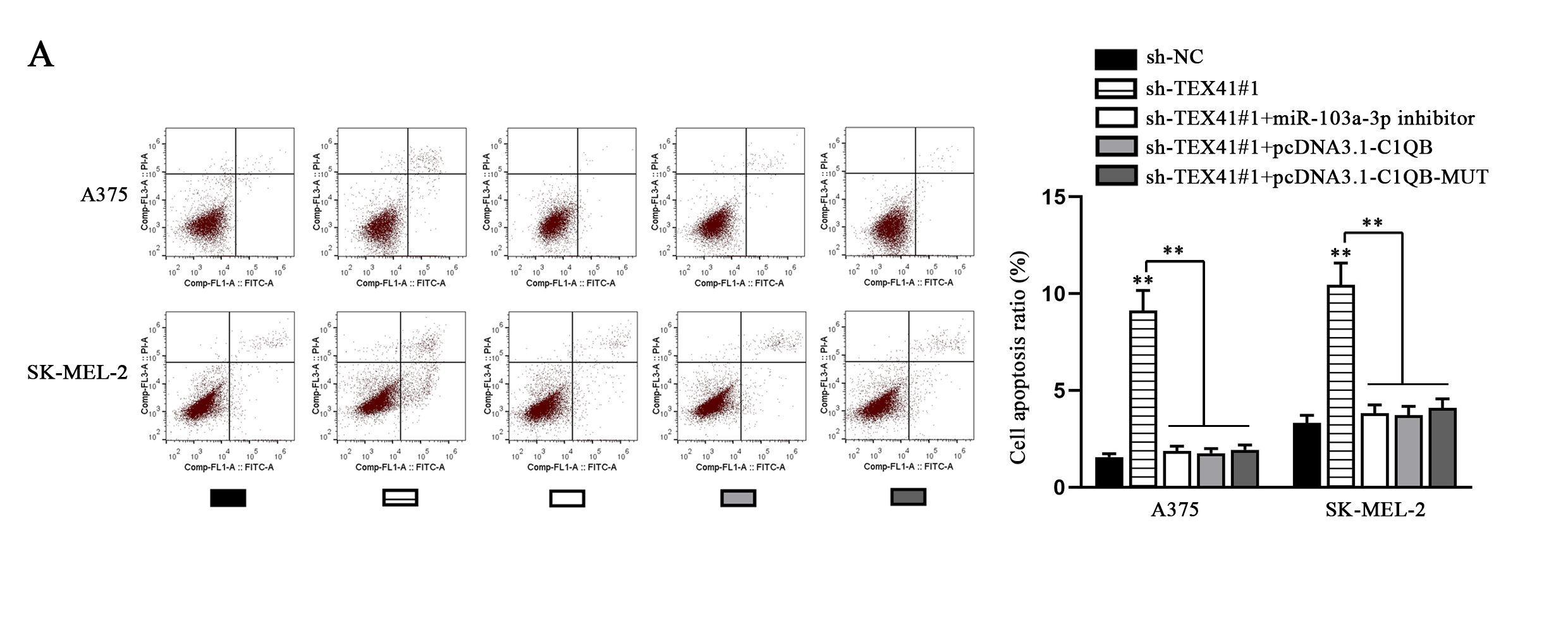

Supplement: Supplementary file 2 — Additional file 2: Figure S2. (A) Apoptosis of melanoma cells was measured by flow cytometry assays. ** P < 0.01. [file 12885_2021_9039_MOESM2_ESM.tif]

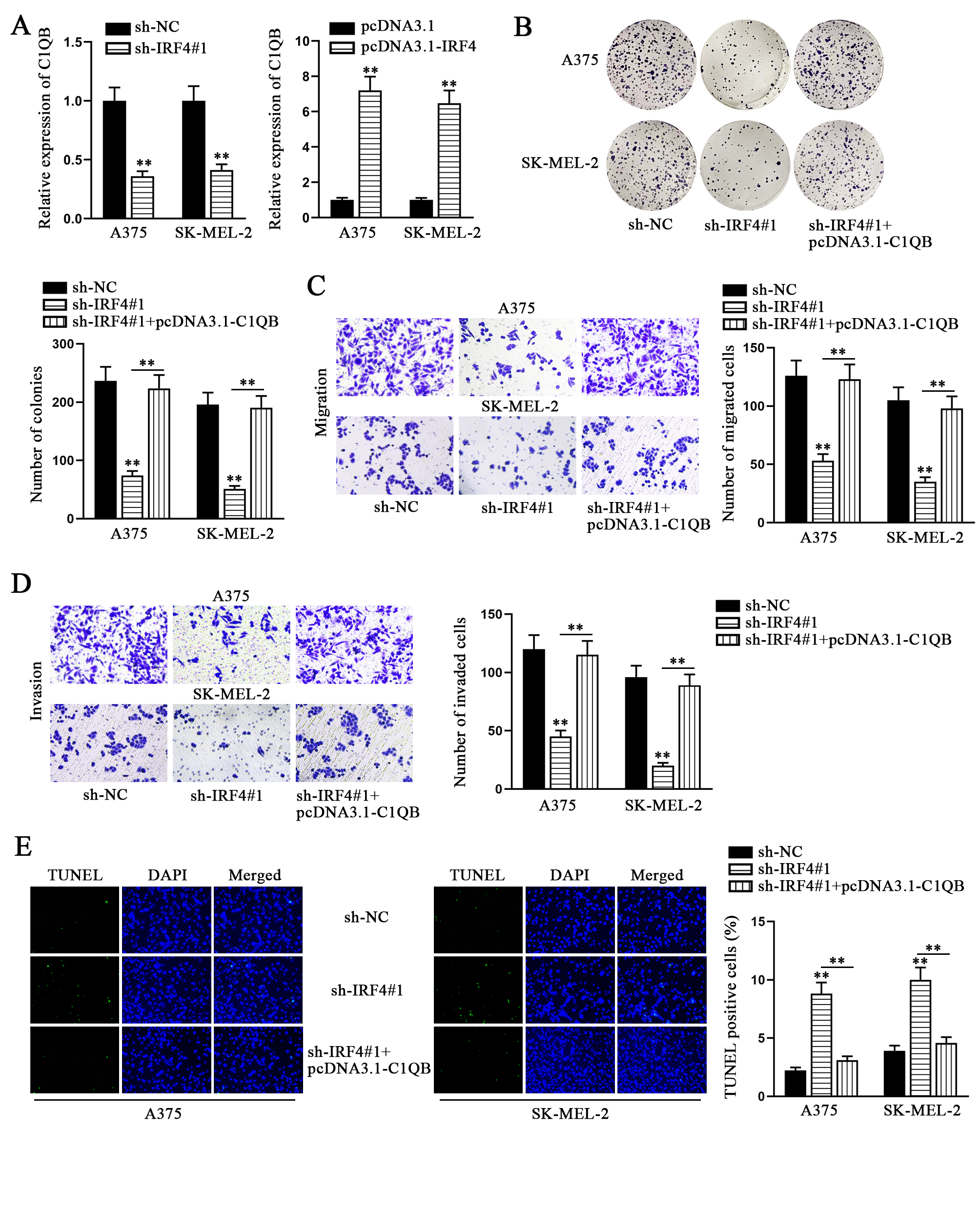

Supplement: Supplementary file 3 — Additional file 3: Figure S3. (A) The expression of C1QB in melanoma cells was quantified via qRT-PCR after IRF4 depletion/overexpression. In rescue assays, three groups were set: sh-NC, sh-IRF4#1 and sh-IRF4#1+pcDNA3.1-C1QB. (B) Colony formation assay was carried out to evaluate proliferation of melanoma cells under different conditions. (C-D) Cell migratory and invasive abilities were analyzed in transwell assays. (E) Cell apoptosis was detected via TUNEL assay. ** P < 0.01. [file 12885_2021_9039_MOESM3_ESM.tif]
